# Role of Surgical Apgar Scores in Predicting the Outcomes of Hollow Viscus Perforation: A Prospective Study in a Tertiary Care Facility in Tripura, India

**DOI:** 10.7759/cureus.88812

**Published:** 2025-07-26

**Authors:** Gowtham K Gudimetla, Sambit Debbarman, Diptendu Chaudhuri, Ranjit Reang, Suryadipta Ghosh, Venkateswarlu Aareddula, Prajnaparmita Saha

**Affiliations:** 1 General Surgery, Agartala Government Medical College, Agartala, IND; 2 Anaesthesiology, Agartala Government Medical College, Agartala, IND; 3 Pharmacology, Agartala Government Medical College, Agartala, IND

**Keywords:** clavien-dindo grading, hollow viscus perforation, postoperative complication, postoperative outcomes, surgical apgar score (sas)

## Abstract

Background

Hollow viscus perforation is a surgical emergency with variable outcomes. The surgical Apgar score (SAS) offers a simple method for risk stratification, but its utility in the patient population is unclear. This study aimed to evaluate the role of SAS in predicting postoperative complications in patients undergoing surgery for hollow viscus perforation.

Methods

This prospective observational study included 106 patients aged 18 years and above who underwent surgery for hollow viscus perforation at a tertiary care center in Tripura, India, between August 2022 and January 2024. The SAS was calculated intraoperatively based on estimated blood loss, lowest mean arterial pressure, and lowest heart rate. Postoperative complications were graded using the Clavien-Dindo classification. The correlation between SAS and complication severity was analyzed.

Results

The mean age of the participants was 41.3 ± 15.2 years, with male predominance (87%, 92/106). Gastric perforation was most common (67.9%, 72/106). The most frequently observed SAS was 6, accounting for 39.6% (42/106) of the cases. A strong negative correlation was found between the SAS and Clavien-Dindo grade (Spearman's ρ = -0.664, p < 0.001), indicating that lower SASs were associated with higher grades of complications.

Conclusion

The SAS demonstrated a significant inverse correlation with postoperative complication severity in patients who had undergone surgery for hollow viscus perforation. The SAS may serve as a simple yet effective tool for predicting outcomes and informing postoperative management in this setting.

## Introduction

Hollow viscus perforation is a surgical emergency and remains one of the more challenging conditions in surgical practice due to its diverse causes and variable outcomes. Consequently, there is a clear need for reliable prognostic tools for this patient group to ensure optimal care delivery and efficient resource utilization. This condition is a major contributor to both morbidity and mortality. Clinical outcomes can vary widely - some patients recover without significant complications, while others may require prolonged intensive care or succumb to their illness [1,2].

Risk scoring systems offer a standardized approach to estimating a patient's likelihood of developing postoperative complications, often taking into account multiple clinical variables, including pre-existing comorbidities. However, the complexity of these systems and their dependence on extensive laboratory data often hinder their use in real-time clinical settings. As a result, they are rarely adopted by surgeons and clinicians at the bedside [3,4].

The surgical Apgar score (SAS) offers a simple, objective, and readily applicable alternative. It is a 10-point intraoperative scoring system based on three readily available parameters: the lowest heart rate, lowest mean arterial pressure, and estimated blood loss. Originally developed for general and vascular surgeries, the SAS has demonstrated predictive value in anticipating postoperative complications and provides a feasible method for early risk stratification without the need for additional investigations [5].

Among the various modern risk assessment tools available for surgical patients, the SAS stands out as a practical and easily implementable option. Its simplicity and reliance on intraoperative hemodynamic parameters make it suitable for routine use. The SAS offers an immediate and objective method for estimating the likelihood of postoperative complications at the conclusion of surgery. By evaluating parameters such as the lowest heart rate, lowest mean arterial pressure, and estimated blood loss, the SAS enables the surgical team to promptly stratify patients into three distinct risk categories: low, moderate, and high. Evidence suggests that patients classified as high risk (SAS: 0-4) have a significantly elevated chance - up to 16 times higher - of developing major postoperative complications compared to those in the low-risk group (SAS: 7-10) [5,6].

However, the relevance of the SAS remains insufficiently explored in resource-limited tertiary care settings, particularly in regions such as Northeast India, including the state of Tripura. Therefore, the present study aims to evaluate the utility of the SAS in predicting postoperative complications among patients with hollow viscus perforation who had undergone exploratory laparotomy.

## Materials and methods

Study design, setting, and population

This hospital-based observational study was conducted in the Department of Surgery at Agartala Government Medical College and GBP Hospital (AGMC & GBPH) between August 2022 and January 2024. The study population consisted of all patients aged 18 years and above who underwent surgical intervention for hollow viscus perforation during the study period.

Selection criteria

All patients aged 18 years and above diagnosed with hollow viscus perforation and who had undergone surgical intervention were included in the study. Exclusion criteria comprised those who declined to provide informed consent for participation, pregnant women, and patients diagnosed with inflammatory bowel disease (IBD) or gastrointestinal malignancies based on postoperative biopsy reports. Patients with IBD and gastrointestinal malignancies were excluded from the study, as these conditions are commonly associated with immunosuppression - either from the underlying pathology or its treatment - which may independently influence postoperative outcomes. Pregnant women were also excluded, given the distinct physiological changes during pregnancy that significantly alter surgical risk and postoperative recovery, warranting separate consideration.

Sample size and sampling

**Figure 1:**
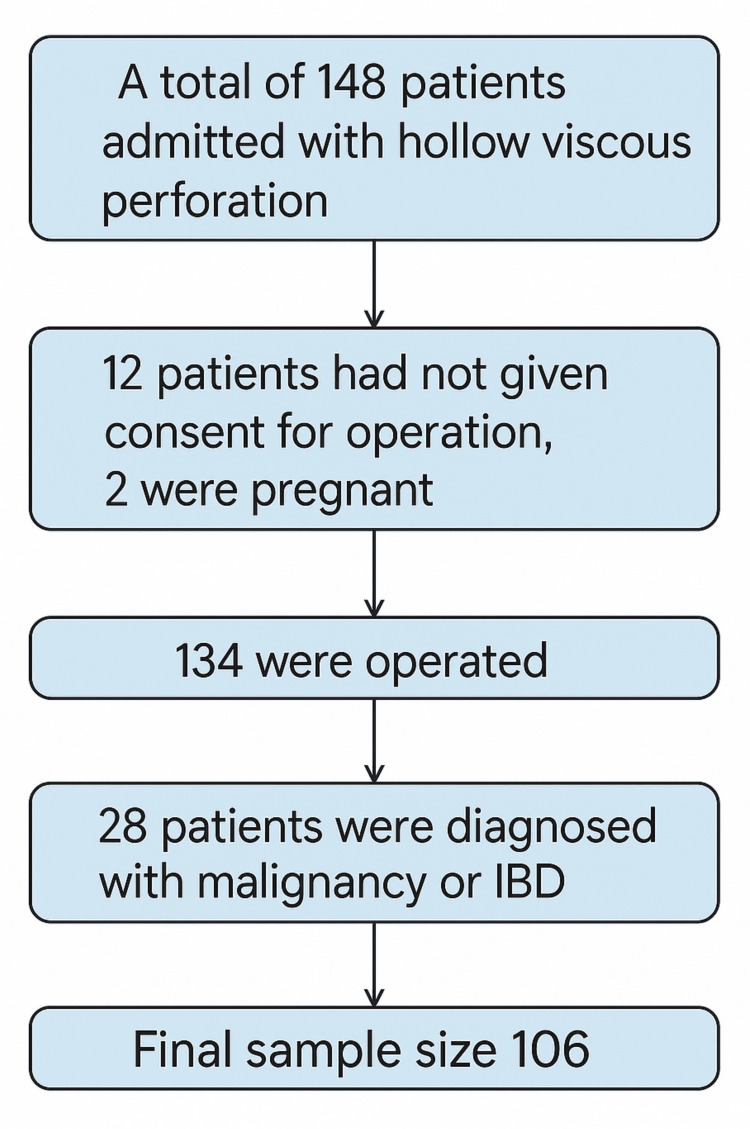
Schematic flow chart of sample selection

The required sample size was calculated to detect a minimum expected correlation coefficient of ρ=0.30 (two-tailed), with a significance level of α=0.05 and power (1 - β) of 0.80. Using Fisher’s z-transformation approach, the estimated minimum required sample size was 84 participants. This calculation was performed using G*Power (version 3.1; The G*Power Team, Germany) [7]. However, we had recruited all eligible participants considering the inclusion and exclusion criteria, and a total of 106 patients were included as the final sample size. A consecutive sampling technique was employed for participant selection. A schematic flow chart of sample selection is depicted in Figure 1.

Study procedure and data collection

Patients who fulfilled the inclusion criteria were enrolled in the study following a comprehensive clinical evaluation, including detailed history-taking, physical examination, radiological investigations, and baseline biochemical investigations. Data were collected using a semi-structured proforma, which included demographic information, clinical details related to gastrointestinal perforation, intraoperative findings, surgical procedures performed, and variables required to compute the SAS, along with postoperative complications.

The intraoperative SAS was calculated based on three parameters: estimated blood loss (EBL), the lowest intraoperative mean arterial pressure (MAP), and the lowest intraoperative heart rate (HR). The EBL was estimated using the following formula [8]: 

$EBL=EBV&times;(Hi&minus;Hf)/{(Hi+Hf)/2}+(500&times;Tu)$

Here, EBV is the estimated blood volume, calculated as body weight in kilograms × 70 mL/kg; Hi is preoperative hemoglobin (g/dL); Hf is postoperative hemoglobin measured approximately 24 hours after surgery (g/dL); and Tu is the number of transfused blood units.

Scoring for EBL was assigned as follows: >1,000 mL = 0 points; 601-1,000 mL = 1 point; 101-600 mL = 2 points; and ≤100 mL = 3 points. The lowest recorded MAP during surgery was scored as follows: <40 mmHg = 0 points; 40-54 mmHg = 1 point; 55-69 mmHg = 2 points; and ≥70 mmHg = 3 points. Similarly, the lowest intraoperative HR was scored as follows: >85 bpm = 0 points; 76-85 bpm = 1 point; 66-75 bpm = 2 points; 56-65 bpm = 3 points; and ≤55 bpm = 4 points. The SAS was computed as the sum of these three components, yielding a score ranging from 0 to 10.

Postoperatively, patients were observed for 30 days to monitor for the development of early complications. These complications were classified and graded using the Clavien-Dindo classification system [9,10].

Data analysis

Data were entered into a Microsoft Excel spreadsheet (Microsoft® Corp., Redmond, WA) and analyzed using Statistical Product and Service Solutions (SPSS, version 26.0; IBM SPSS Statistics for Windows, Armonk, NY). Continuous variables were summarized as mean ± standard deviation, while categorical variables were expressed as frequencies and percentages. Spearman’s rank correlation was used for inferential statistical analysis. A p-value of <0.05 was considered indicative of statistical significance.

## Results

The mean age of the patients was 41.3 ± 15.2 years, with ages ranging from 18 to 72 years. A majority of the patients were male (n = 92, 87%), while females accounted for 13% (n = 14). The most frequently observed site of perforation was the stomach, identified in 72 patients (67.9%). This was followed by ileal perforations in 15 cases (14.2%) and duodenal perforations in 11 cases (10.4%). Jejunal involvement was noted in five patients (4.7%), whereas colonic perforation was the least common, observed in three patients (2.8%). The majority of these perforations were spontaneous in origin, accounting for 72.6% (77/106) of cases, whereas the remaining 27.3% (29/106) were attributed to traumatic causes.

**Figure 2:**
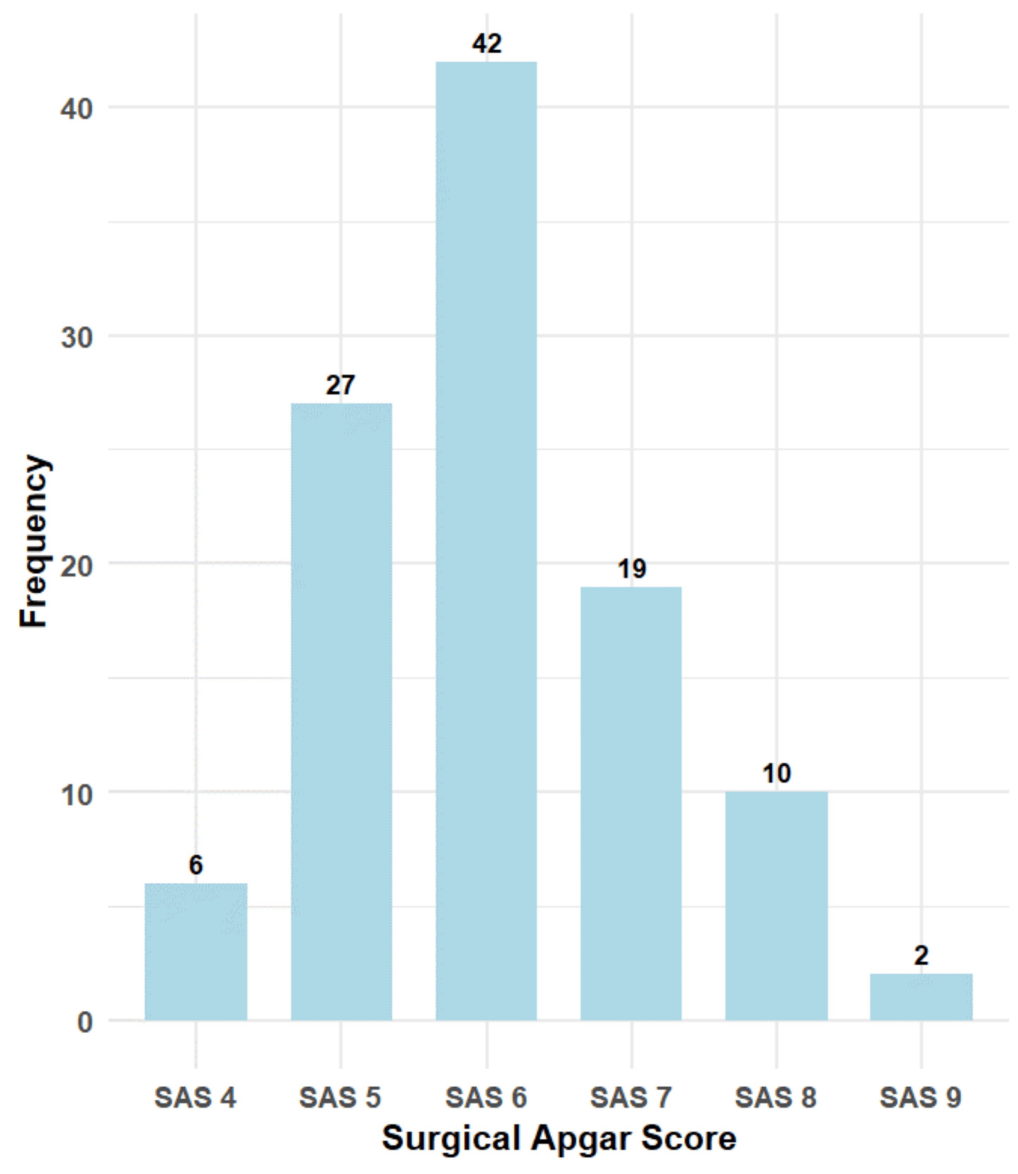
Distribution of surgical Apgar scores among participants

The most frequently observed SAS was SAS 6, recorded in 42 cases (39.6%), followed by SAS 5 in 27 cases (25.5%) and SAS 7 in 19 cases (17.9%). SAS 8 was noted in 10 cases (9.4%), SAS 4 in six cases (5.7%), and the least frequent was SAS 9, seen in only two cases (1.9%) (Figure 2).

Several postoperative complications were observed. Respiratory failure occurred in 17 patients (16.0%) and wound infection in 10 patients (9.4%), and death was reported in 20 patients (18.9%) (Table 1). Complications were graded according to the Clavien-Dindo classification system. The majority of patients (69/106, 65.1%) experienced Grade I complications, while 17 patients (16.0%) had Grade IVA complications, and 20 patients (18.9%) had Grade V complications.

**Table 1:** Distributions of postoperative complications among participants (n=106)

Complications	Frequency	Percentage
No complications	59	55.7%
Respiratory failure	17	16.0%
Wound infection	10	9.4%
Death	20	18.9%

**Figure 3:**
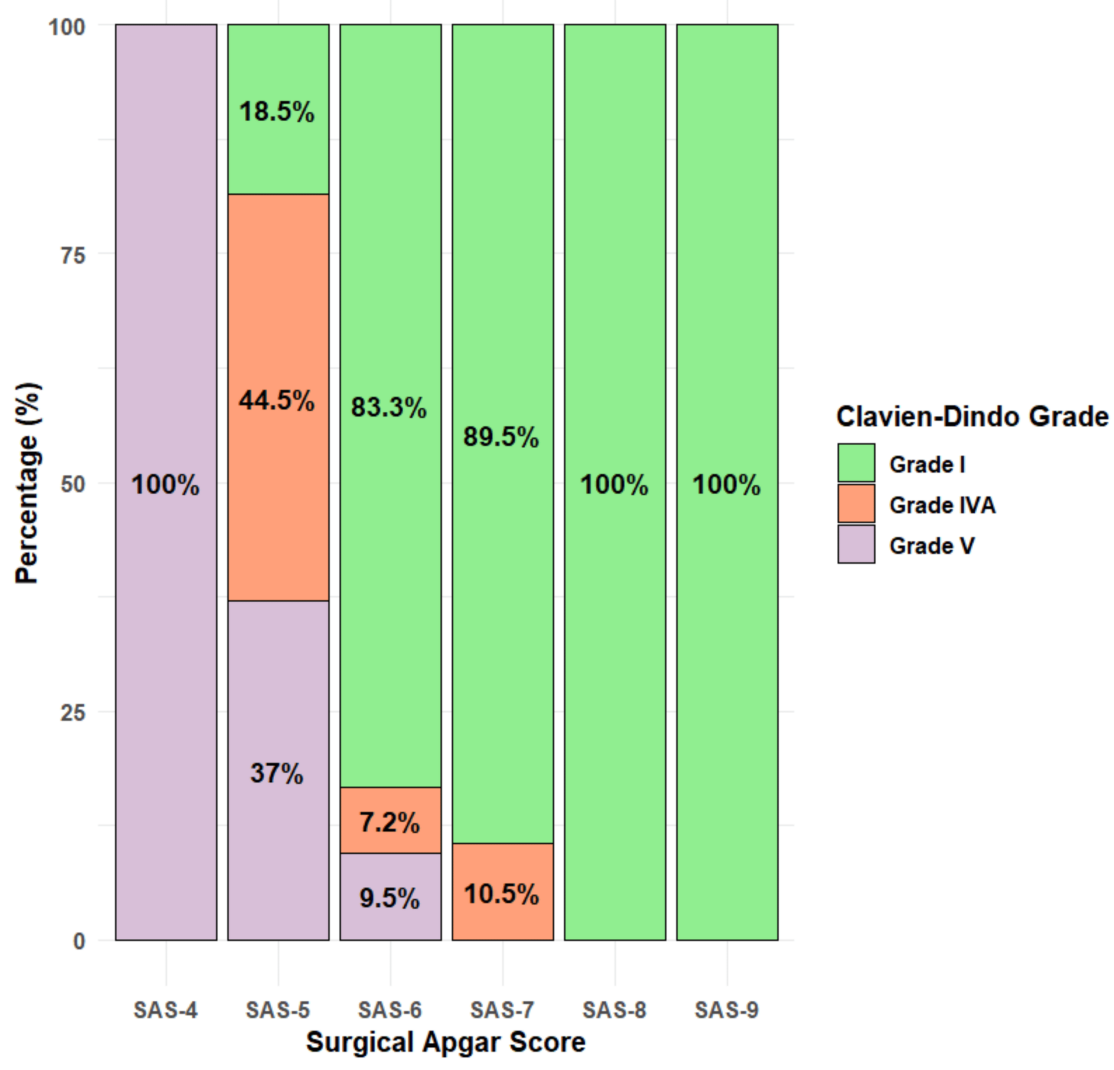
Distribution of complication across surgical Apgar scores

Among patients with the SAS of 4, all six (100%) experienced Clavien-Dindo Grade V complications. For those with SAS 5, 10 (37.0%) had Grade V complications, 12 (44.4%) had Grade IVA, and five (18.5%) had Grade I. Among patients with SAS 6, four (9.5%) had Grade V, three (7.1%) had Grade IVA, and 35 (83.3%) had Grade I complications. For SAS 7, two (10.5%) patients had Grade IVA, and 17 (89.5%) had Grade I complications. All patients (100%) with SAS 8 (10 cases) and SAS 9 (two cases) experienced only Grade I complications. Notably, higher SAS was associated with lower complication grades (Figure 3).

Spearman’s rank correlation analysis revealed a statistically significant, strong negative correlation between the SAS and Clavien-Dindo Grade (ρ = −0.664, p < 0.001, n = 104). This indicates that lower SASs are associated with higher Clavien-Dindo Grades, suggesting a greater severity of postoperative complications.

## Discussion

This study was done to determine the relevance of the SAS in predicting major postoperative complications in patients who had undergone surgery for hollow viscus perforation. Our study findings depict a clear inverse correlation between the SAS and the severity of postoperative complications, which was measured by the Clavien-Dindo grading system.

In the present study, the mean age of participants was 41.3 ± 15.2 years, with a notable male predominance (87%). This demographic pattern is consistent with previous literature. For instance, Tripura et al. reported a mean age of 46.5 ± 5.2 years, and 83.3% were male patients in their cohort [11]. Similarly, Hota et al. observed that most of their cases occurred in the 41-60 years age group, with males accounting for 72.1% of the study population [12]. The consistent male predominance across studies can be attributed to several behavioral and physiological factors. Males are more frequently exposed to known risk factors for gastrointestinal (GI) perforations, including a higher prevalence of peptic ulcer disease, greater use of nonsteroidal anti-inflammatory drugs (NSAIDs), and significantly higher rates of smoking - each contributing to mucosal injury and increased susceptibility to perforation. In line with these findings, Chandran et al. also documented a higher incidence of hollow viscus perforation among male patients [2]. Regarding the anatomical site of perforation, the stomach was the most frequently involved organ in our study, accounting for 67.9% of cases. This was followed by ileal (14.2%) and duodenal (10.4%) perforations. These findings highlight the dominance of upper GI tract involvement, particularly peptic ulcer-related perforations. Similar trends have been reported in the literature. Chandran et al. documented that gastric and duodenal ulcer perforations constituted the most common cause of hollow viscus perforations in their study population, comprising approximately 51% of cases [2]. This reflects a persistent burden of acid-peptic disease in emergency surgical settings. Conversely, some studies report different patterns of site distribution. For instance, Yadav et al. reported a higher frequency of ileal perforation (39.1%), followed by duodenal (26.4%) and gastric (11.5%) perforations [13]. These variations may be attributed to regional differences in disease epidemiology, healthcare access, diagnostic delays, and dietary or lifestyle factors that influence the prevalence of specific GI pathologies.

In the present study, the most frequently observed SAS was 6, accounting for 39.6% of the cases, followed by a score of 5 (25.5%) and 7 (17.9%). This distribution highlights that a significant proportion of patients fell within the moderate-risk category. As per established literature, the SAS has been categorized into three broad risk groups: low risk (SAS: 8-10), moderate risk (SAS: 5-7), and high risk (SAS: 0-4) [14-16]. In our cohort, the majority of scores clustered within the moderate range, which is consistent with findings from previous studies on surgical emergencies, where patients who underwent emergency GI procedures often exhibited intermediate SAS values due to their acute physiological status and intraoperative challenges [14]. Our analysis revealed a statistically significant and strong negative correlation between the SAS and Clavien-Dindo grades of postoperative complications (Spearman’s ρ = -0.664, p < 0.001). This indicates that, as the SAS decreases, the severity of postoperative complications increases. Specifically, patients with SAS ≤5 were more likely to experience high-grade complications (Grades IV and V), including life-threatening events and mortality. Conversely, those with higher SAS (≥7) predominantly suffered from minor complications (Grade I), reflecting a more favorable postoperative course. These findings are in concordance with earlier studies. Shah et al. also reported that patients with lower SAS were more prone to severe postoperative complications compared to those with higher scores, supporting the utility of SAS in perioperative risk stratification [17]. Furthermore, Gawande et al., who originally proposed the SAS, demonstrated that patients with SAS ≤4 had significantly increased risks of major complications and mortality. In their study, the risk ratio for major complications and deaths among patients with a score of 9 or 10 compared to those with a score ≤4 was 16.1 (95% confidence interval (CI): 7.6-34.0; p < 0.0001), emphasizing the prognostic value of SAS [6]. Similarly, Tracy et al. underscored the predictive power of the SAS in emergency GI surgeries. Their study showed that an SAS of ≤4 was strongly associated with higher incidences of respiratory failure, septic shock, and overall postoperative morbidity (p < 0.01) [18].

Patients undergoing major abdominal surgery are inherently at increased risk for adverse peri- and postoperative events. The findings of our study highlight the clinical significance of the SAS as a simple, intraoperative tool for predicting postoperative complications, morbidity, and mortality in emergency surgical settings. Incorporating the SAS into routine surgical assessment can enhance postoperative risk stratification, inform decisions regarding the intensity of postoperative monitoring, and facilitate clearer communication with patients and their families about prognosis and expected outcomes. Given its ease of use, objectivity, and prognostic value, the SAS represents a valuable adjunct to standard perioperative assessment and may be considered for integration into routine intraoperative practice to support clinical decision-making and quality improvement efforts.

Strengths and limitations

This study is strengthened by using standardized scoring systems, particularly the Clavien-Dindo classification for postoperative complications, which enhances the consistency and objectivity of outcome assessment. Nonetheless, a few limitations should be acknowledged. The single-center design may restrict the generalizability of findings to broader populations. Although standardized tools were employed, the potential for observer bias cannot be entirely excluded. The follow-up period was limited to 30 days, which may have resulted in underreporting of late-onset complications. Moreover, the study did not include a comparison with other established risk scoring systems to evaluate the relative performance of the SAS.

## Conclusions

The present study revealed a significant inverse correlation between the SAS and the severity of postoperative complications in patients who had undergone surgery for hollow viscus perforation. Lower SAS scores were correlated with higher grades of complications as classified by the Clavien-Dindo system. These findings substantiate the SAS as a straightforward yet effective tool for predicting postoperative outcomes in this patient cohort. The simplicity and objectivity of the SAS render it a valuable complement to standard perioperative assessments. Integrating the SAS into routine surgical practice could enhance risk stratification, inform postoperative care decisions, and improve communication with patients and their families regarding anticipated outcomes. Further research to validate these findings in larger and more diverse populations is warranted.
